# Frozen in Place: Proximity Labeling Maps Glial Interactomes Across Cell States

**DOI:** 10.1002/glia.70216

**Published:** 2026-08-18

**Authors:** João Baltar, Rebecca Abati, Luísa Florido, Matthew G. Holt

**Affiliations:** ^1^ Synapse Biology Group Instituto de Investigação e Inovação em Saúde (i3S), University of Porto Porto Portugal; ^2^ Faculdade de Medicina da Universidade do Porto Porto Portugal; ^3^ Instituto de Biologia Molecular e Celular, Universidade do Porto Porto Portugal; ^4^ Instituto de Ciências Biomédicas Abel Salazar Porto Portugal

**Keywords:** cell–cell communication, glial cells, protein networks, proteomics, proximity labeling

## Abstract

Glial cells, including radial glia, oligodendrocyte precursor cells (OPCs), oligodendrocytes, astrocytes, and microglia, are active and dynamic regulators of central nervous system (CNS) development, homeostasis, and disease. Through extensive interactions with neurons, other glial populations, and the vasculature, they form highly specialized communication networks that are essential for normal brain function. While transcriptomic approaches have revealed extensive glial heterogeneity and enabled the prediction of putative signaling networks, a critical challenge remains in validating and translating these findings at the level of distinct protein complexes existing both within and between the various glial cell types. This is largely due to the fact that traditional proteomic technologies lack spatial resolution and/or fail to capture protein interaction networks. Proximity labeling (PL) has emerged as a powerful strategy to overcome these limitations by enabling cell‐type‐specific mapping of protein networks and subcellular proteomes, with spatial and temporal precision. Emerging studies have applied PL enzymes, such as BioID, TurboID, and HRP, across diverse glial populations, starting to uncover protein networks supporting their interactions with neurons and vascular elements, allowing metabolic support, maintenance of microenvironment homeostasis and cell–cell communication (including synaptic modulation). In this review, we summarize the main PL enzymes, discuss key studies across different glial cell types, and examine the technical challenges and future perspectives of applying PL to investigate glial biology. By complementing transcriptomic data with spatially resolved proteomic insights, PL provides a unique opportunity to deepen our understanding of glial cell biology in health and disease.

## Graphical abstract

- Proximity labeling enables cell‐type‐specific and spatially resolved mapping of glial protein networks.
- Proximity labeling approaches provide insights into glial function under physiological and pathological conditions.

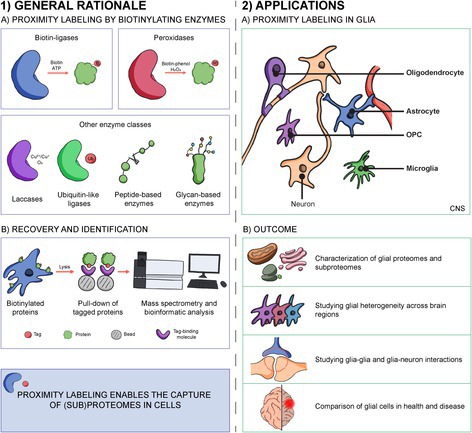

## 1 Introduction

The mammalian brain is a highly complex organ whose function relies on precise and dynamic communication between multiple cell types. Although neurons have traditionally been seen as the main drivers of brain function, glial cells, which constitute around 50% of the brain, are now considered active contributors to neural circuit formation, function, and homeostasis (von Bartheld et al. 2016).

**FIGURE 1:**
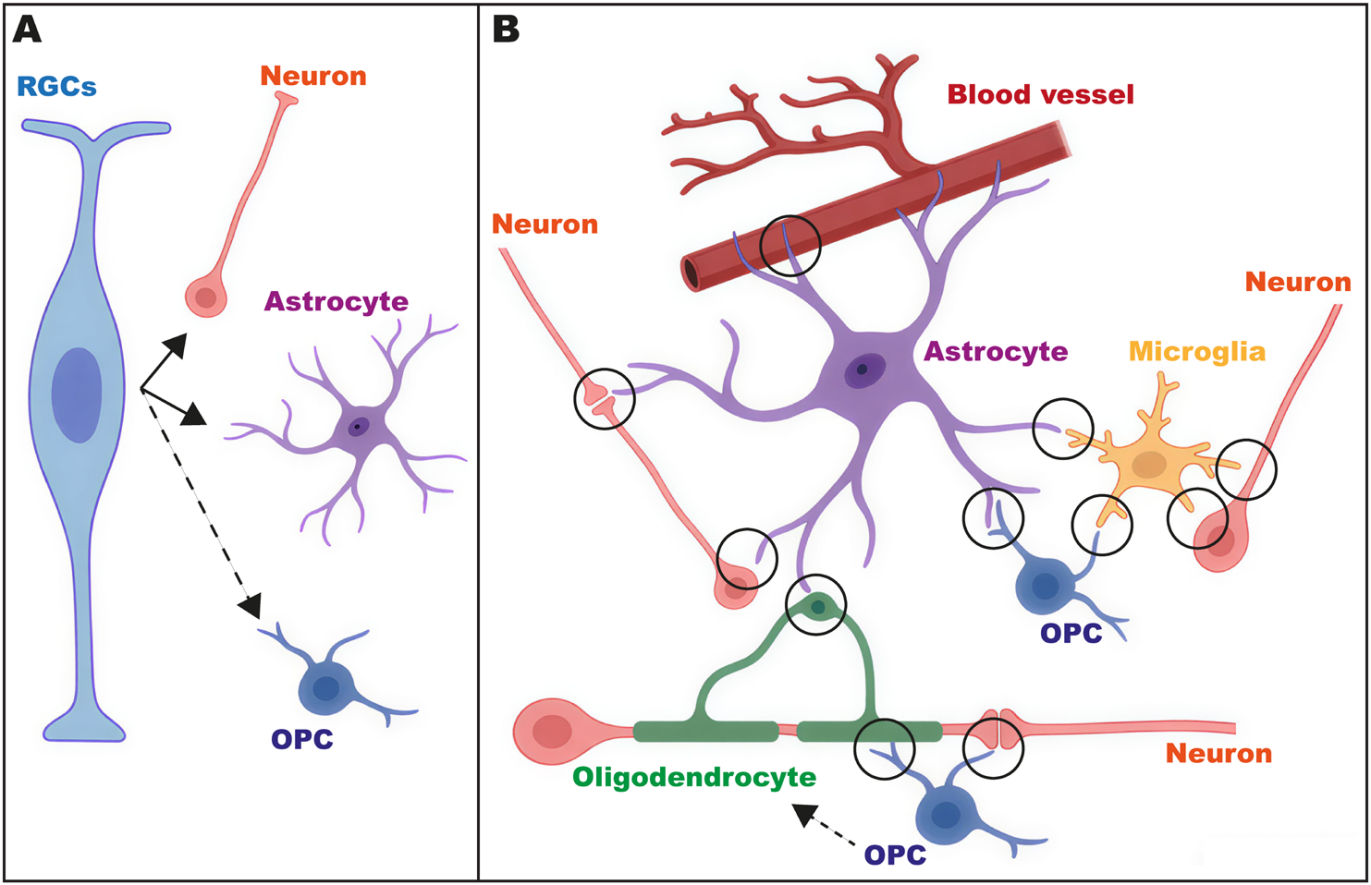
Lineage and interactions of glial cells in the central nervous system. (A) Radial glial cells (RGCs) act as key progenitors in the developing central nervous system (CNS) and give rise to multiple neural and glial lineages, including neurons, astrocytes, and oligodendrocyte precursor cells (OPCs). (B) Schematic representation of the complex communication networks established between glial cells, neurons, and vascular structures within the CNS (depicted with black circles). Created with Biorender.com, modified from Allen and Lyons (2018).

Within the central nervous system (CNS), glial cells can be divided into radial glia cells (RGCs), which are the embryonic precursors that eventually give rise to the oligodendrocyte progenitor cells (OPCs), oligodendrocytes and astrocytes that populate the adult CNS—with microglia, in contrast, deriving from yolk‐sac progenitors, and colonizing the CNS during early embryogenesis (Allen and Lyons 2018; Zuchero and Barres 2015). These different glial populations engage in extensive interactions with neurons, other glial cells, and the vasculature, modulating brain development and function across multiple scales (Figure 1).

During development, RGCs also generate most neurons, as well as glia, and provide a scaffold that guides neuronal migration and establishes the early cellular organization of the brain (Figure 1A) (Hatanaka et al. 2019; Noctor et al. 2001). Given their principal role as progenitors, RGCs are absent from the mature CNS.

OPCs, once regarded solely as intermediate progenitors, also interact with both neurons and other glial cells (Figure 1B). They receive inputs from multiple neuronal types and influence neural circuit remodeling, while also acting as a proliferative progenitor pool that differentiates into myelinating oligodendrocytes (Benarroch 2023). Furthermore, OPCs engage in bidirectional interactions with astrocytes and microglia at synapses, forming part of a complex glial network that modulates synaptic development and function (Liu et al. 2023). Upon differentiation, oligodendrocytes not only myelinate axons but also secrete factors that are taken up by neurons, supporting axonal transport, energy homeostasis, and resilience to stress (Frühbeis et al. 2020).

Astrocytes, the most abundant glial cell type in the mammalian brain, interact not only with neurons and other glial cells but also with the vasculature (Figure 1B) (Endo et al. 2022). Through their endfeet, they contribute to the formation and maintenance of the blood–brain barrier (BBB), while extending perisynaptic processes (PAPs) that closely associate with synapses. These interactions are mediated either via cell adhesion molecules, such as neuronal cell adhesion molecule (NrCAM), or through secreted factors, including thrombospondins (TSPs) and neurocan (NCAN) (Christopherson et al. 2005; Irala et al. 2024; Takano et al. 2020).

Similarly, microglia, the resident immune cells of the CNS, interact with neurons and other glial populations to regulate synaptic refinement, immune surveillance, and tissue homeostasis (Figure 1B). Recent studies have proposed microglia may even communicate directly with their targets through the use of tunneling nanotubes that allow bidirectional transfer of cellular components (Chakraborty et al. 2023).

Collectively, these examples underscore that proper brain function relies on precise communication between different cell types.

Individual glia types are themselves highly heterogeneous across brain regions, showing differences based on developmental stage, sex, and species (Seeker et al. 2023). This heterogeneity manifests at multiple levels, including molecular signatures, morphological properties, physiological functions, and response to injury/disease (Oliveira et al. 2026). At the molecular level, distinct glial subtypes differ in their expression of surface markers, receptors, and transporters, as well as in their intracellular signaling pathways, which underlie their capacity to respond to inputs from other cell types with an appropriate response. Morphologically, glial cells display remarkable diversity in their branching complexity, territorial domains, and contacts with synaptic and vascular elements, reflecting functional specialization across brain regions and cell state. This baseline heterogeneity likely underlies the differential response to injury and disease (“reactivity”) observed across glial types at the molecular, cellular and physiological levels (Pestana et al. 2020).

Over the past decade, glial heterogeneity has been most extensively investigated at the transcriptome level using bulk RNA‐seq, single‐cell (scRNA‐seq), and single‐nucleus (snRNA‐seq) approaches, fundamentally advancing our understanding of cell diversity and cell state (Batiuk et al. 2020; Hammond et al. 2019; Zeisel et al. 2015). These studies revealed that glial cells do not consist of uniform populations but do, in fact, comprise multiple transcriptionally distinct subtypes. Moreover, transcriptomic datasets are increasingly leveraged to infer putative cell–cell communication networks based on ligand‐receptor expression profiles, using computational frameworks, such as CellChat, which are widely applied to predict signaling interactions between neurons and glial cells (Jin et al. 2025). While informative, transcriptomics‐based studies have a number of well‐known limitations. Foremost among these is the apparent disparity between transcriptome and proteome, which studies suggest may be significant in the CNS (de Sousa Abreu et al. 2009; Maier et al. 2009; Sharma et al. 2015). Such disparities can be caused by post‐transcriptional regulation of transcript levels, including at the levels of mRNA stability, alternative splicing, and translational efficiency (Zhao et al. 2017). Furthermore, proteins undergo post‐translational modifications (PTMs), such as phosphorylation, ubiquitination, and glycosylation, that critically regulate protein activity, subcellular localization, and interaction dynamics (Zhong et al. 2023). This disconnect is particularly relevant in the CNS, where additional regulatory mechanisms, including RNA‐binding protein activity, local mRNA translation at synapses, and brain‐enriched miRNA networks, further decouple transcript and protein dynamics. Moreover, spatially and temporally restricted protein complexes mediate key signaling events that cannot be inferred from transcriptomic data alone (Yang et al. 2015).

The earliest attempts to study protein interactions in glial cells largely used co‐immunoprecipitation (co‐IP) methods (Weerth et al. 2007). Co‐IP enables the identification of protein–protein interactions through antibody‐mediated capture of target proteins and their interacting partners, following detergent‐based cell lysis. This step is frequently performed with harsh ionic detergents to maximize protein recovery with low non‐specific background binding; however, this type of protocol inevitably dissociates *bona fide* weak or transient interactions, which are thought to play key roles in regulating dynamic cellular processes, biasing discovery towards stable protein complexes. On the other hand mild, non‐denaturing lysis buffers can help to preserve some protein complexes at the risk of increased background. Inevitably, however, all co‐IP strategies typically lack spatial resolution, unless combined with some form of subcellular fractionation. In addition, co‐IP depends on the availability of high‐quality antibodies (Tan and Yammani 2022). These limitations are particularly problematic in the brain, where signaling often occurs within highly specialized and spatially restricted domains such as synapses, perivascular astrocyte endfeet, or fine glial perisynaptic processes.

Alternative approaches, such as yeast two‐hybrid (Y2H) assays, have also been used to map protein interaction networks, but present their own distinct limitations. Y2H relies on the overexpression of “bait” and “prey” proteins in a yeast system, which does not recapitulate endogenous protein levels, PTMs, or the cellular environment of mammalian glial cells (Brückner et al. 2009). Furthermore, Y2H is inherently limited to pairwise interactions and cannot capture the complexity of multi‐protein complexes. Notably, its application to questions of glial biology remains limited, and interactions identified by this method may not reflect those occurring under physiological conditions in the CNS.

Proximity labeling (PL) has addressed many of these limitations by enabling the selective labeling of proteins in close spatial proximity within intact cells and tissues, both in vitro and in vivo. This approach allows the detection of stable as well as transient interactions within their native physiological context (Chen and Perrimon 2017). Importantly, PL can be combined with genetic strategies, including viral vector‐mediated delivery or transgenic mouse lines, to achieve cell‐type‐specific expression (Soto et al. 2023, 2024).

In the context of glial biology, PL has enabled the systematic mapping of protein networks that shape cell identity, function, and interactions with neighboring cells. Although its application remains more limited than in neuronal studies, accumulating evidence indicates that it can reveal distinct glial proteomes, resolve subcellular protein distributions, and capture dynamic molecular interactions under both physiological and pathological conditions (Sunna et al. 2023).

This review compares the main PL enzymes, summarizes published studies applying PL in glial biology, with a particular focus on astrocytes—the glial cell type most extensively studied using these approaches—and offers a future perspective for the field, focusing on a discussion of the opportunities and challenges associated with applying this technique to glial cell biology.

## 2 PL Using Biotinylating Enzymes

Biotinylating enzymes can be divided into two main categories based on the enzyme activity: biotin‐ligases and peroxidases. In recent years, additional variants have been developed, including laccases, ubiquitin‐like ligases, peptide‐based and glycan‐based PL approaches. While these newer enzymes expand the methodological repertoire, they will only be mentioned briefly here, as no studies to date have applied them to glial cells.

Importantly, different enzymes display distinct kinetics and, therefore, require different labeling times to achieve efficient protein tagging. In addition, they also exhibit different optimal temperatures, effective labeling radii, and amino acid preferences, which may introduce biases in the identified protein sets (e.g., lysine residues for BioID and tyrosine/tryptophan residues for APEX2). Moreover, the subcellular localization of the enzyme, and whether it is membrane anchored or cytosolic, can strongly affect labeling outcomes (e.g., expression in the cytosol versus the lumen of the endoplasmic reticulum) (Kim et al. 2021), underscoring the need for appropriate controls to assess background biotinylation.

Despite these limitations, PL approaches offer clear advantages, including efficient recovery of membrane‐associated proteins under harsh lysis conditions and simple streptavidin‐based pull‐downs. Where feasible, the use of complementary systems or independent validation strategies can further strengthen data interpretation. For example, direct comparisons of different PL enzymes (e.g., APEX2 vs. TurboID) reveal distinct yet complementary labeling profiles across compartments (Battison et al. 2025). The major issue with PL is that it generally tends to struggle to identify *bona fide* interactors from proteins merely proximate to the enzyme. Nevertheless, when combined with targeting to specific cellular compartments, PL still provides unprecedented insights into the subcellular molecular machineries driving cell function.

### 2.1 Biotin‐Ligases

**FIGURE 2:**
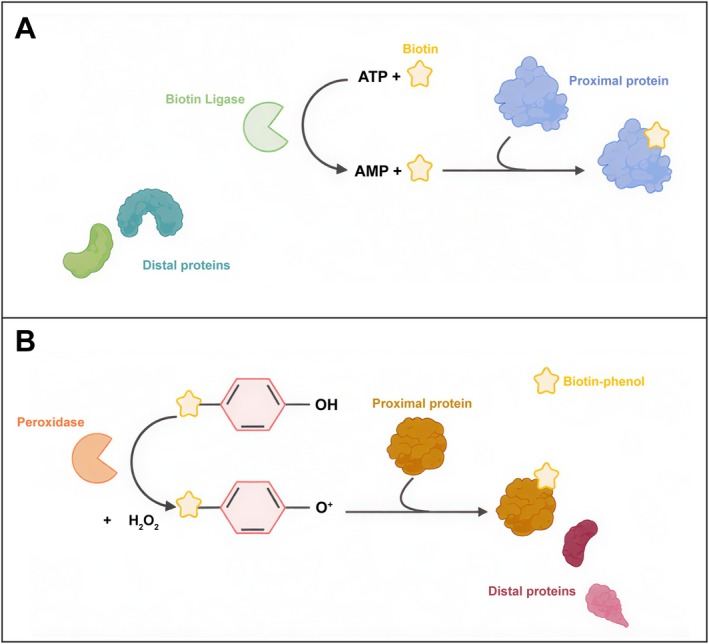
Schematic overview of proximity labeling strategies. (A) Schematic representation of proximity‐dependent protein labeling by biotin‐ligases. The enzyme activates biotin using ATP, forming a reactive biotin‐AMP intermediate that covalently attaches to lysine residues on neighboring proteins, while distant ones remain unmodified. (B) Schematic representation of proximity‐dependent protein labeling by peroxidases. In the presence of H_2_O_2_, the enzyme oxidizes biotin‐phenol to a reactive radical that covalently tags neighboring residues. Created with Biorender.com.

Biotin‐ligase‐based PL relies on engineered enzymes, such as BioID, BioID2, microID, microID2, ultraID, TurboID, miniTurboID, AirID and BASU, which catalyse the covalent attachment of biotin to lysine residues on nearby proteins. Biotin‐ligases use adenosine triphosphate (ATP) to convert biotin into a reactive intermediate, biotinoyl‐5′‐AMP (Figure 2A). These enzymes generally label proteins within a radius of ~10 nm, with a reaction window that can extend from minutes to several hours, depending on the enzyme (Table 1) (Chen and Perrimon 2017). BioID and BioID2 were the first widely used biotin‐ligases for PL (Kim et al. 2014, 2016; Roux et al. 2012) and are derived from a mutated form of the *Escherichia coli* biotin‐ligase BirA. Both function at physiological temperatures (~37°C) and need extended labeling times (6–24 h) to achieve sufficient signal, making them ideal to detect stable or long‐lasting protein networks. On the other hand, this low catalytic rate limits their ability to capture transient or dynamic processes, and prolonged labeling can increase background biotinylation (Table 1). Of note, BioID2 is significantly smaller than BioID, enabling more selective targeting of fusion proteins, due to reduced steric hindrance.

**TABLE 1:** Summary of commonly used PL enzymes.

Enzyme activity	Enzyme	Labeling time	Approximate labeling radius	Optimal labeling temperature (°C)	Advantages	Limitations	References
Biotin‐ligase	BioID	6–24 h	10 nm	Above 37	− Uses a non‐toxic substrate − Stable at higher temperatures	− Reduced enzymatic activity below 37°C − Longer labeling times than peroxidases − Lower catalytic efficiency compared to TurboID	Kim et al. 2014, 2016
	BioID2	12–18 h	10 nm	37–50	− Uses a non‐toxic substrate − Requires a lower biotin concentration than BioID − Stable at higher temperatures − Smaller size compared to BioID giving reduced steric hindrance	− Reduced enzymatic activity below 37°C − Longer labeling times than peroxidases − Lower catalytic efficiency compared to TurboID	Kim et al. 2016
	microID	4–24 h	1–10 nm	37	− Uses a non‐toxic substrate − Small size − Lower background labeling than TurboID	− Reduced activity below 37°C − New enzyme: few published studies for benchmarking − Longer labeling times than peroxidases − Lower catalytic efficiency compared to TurboID	Na et al. 2022
	microID2	2–4 h	10 nm	37	− Uses a non‐toxic substrate − Small size − Lower background labeling than TurboID	− Reduced activity below 37°C − New enzyme: few published studies for benchmarking − Lower catalytic efficiency compared to TurboID − Longer labeling times than peroxidases	Johnson et al. 2022
	ultraID	10–30 min	10 nm	37	− Small size − Uses a non‐toxic substrate − Lower background labeling than TurboID − Rapid kinetics with high specificity	− Reduced activity below 37°C − Lower catalytic efficiency compared to TurboID − New enzyme: few published studies for benchmarking − Longer labeling times than peroxidases	Kubitz et al. 2022
	TurboID	10 min	10–35 nm	37	− Uses a non‐toxic substrate − Higher biotinylation activity than BioID and BioID2 − Robust performance across diverse cell types and organisms	− Less control of labeling window − Can deplete intracellular biotin pools − High activity can increase non‐specific labeling − Longer labeling times than peroxidases	Branon et al. 2018; Chen and Perrimon 2017
	miniTurboID	10 min	10–30 nm	30–37	− Uses a non‐toxic substrate − Smaller size compared to TurboID giving reduced steric hindrance − Higher biotinylation activity than BioID and BioID2 − Lower background labeling than TurboID	− Lower catalytic activity and stability compared to TurboID − Longer labeling times than peroxidases	Branon et al. 2018
	AirID	1–3 h	10 nm	37	− Uses a non‐toxic substrate − Higher biotinylation activity than BioID − Does not self‐biotinylate − Reduced off‐target labeling compared to TurboID	− Reduced activity below 37°C − Slower labeling kinetics than TurboID and miniTurboID − Longer labeling times than peroxidases	Kido et al. 2020; Schaack et al. 2023
	BASU	12–24 h	10 nm	37	− Uses a non‐toxic substrate − Higher biotinylation activity than BioID − Enables the study of RNA‐protein interactions	− Reduced activity below 37°C − Slower labeling kinetics compared to TurboID and miniTurboID − Longer labeling times than peroxidases	Ramanathan et al. 2018; Villaseñor et al. 2020
Peroxidase	APEX	1 min	20 nm	25–37	− Higher temporal resolution compared to biotin‐ligases − Does not require ATP	− Potentially toxic due to the use of H_2_O_2_	Chen and Perrimon 2017; Martell et al. 2012
	APEX2	1 min	20 nm	25–37	− Higher temporal resolution compared to biotin‐ligases − More sensitive and active than APEX − Does not require ATP − Higher catalytic activity than HRP	− Potentially toxic due to the use of H_2_O_2_ − Tends to accumulate in the cytoplasm	Chen and Perrimon 2017; Lam et al. 2015
	APEX3	1 min	20 nm	25–37	− Higher temporal control compared to biotin‐ligases − More sensitive and active than APEX and APEX2 − Does not require ATP − More even cellular distribution (cytoplasmic and nuclear) compared to APEX2	− Potentially toxic due to the use of H_2_O_2_	Becker et al. 2023
	HRP	5–10 min	20–50 nm	25–37	− Higher temporal resolution compared to biotin‐ligases − Does not require ATP	− Potentially toxic due to the use of H_2_O_2_ − Inactive in a reducing environment; reduced versatility compared to APEX2	Chen and Perrimon 2017; Martell et al. 2016
Split systems	Split‐BioID	12–24 h	10 nm	37	− Uses a non‐toxic substrate − Low background − Can be applied in vivo	− Reduced activity below 37°C − Longer labeling times than peroxidases − Assembly depends on the design of the expression system, including linker length and fragment orientation − Lower catalytic activity compared to full‐length BioID	De Munter et al. 2017; Schopp et al. 2017
	Split‐TurboID	30 min–2 h	10–35 nm	30–37	− Uses a non‐toxic substrate − Improved temporal resolution compared to split‐BioID − Can be applied in vivo	− Longer labeling times than peroxidases − Assembly depends on the design of the expression system, including linker length and fragment orientation − Lower catalytic activity compared to full‐length TurboID	Cho et al. 2020
	Split‐AirID	3–6 h	10 nm	37	− Uses a non‐toxic substrate	− Longer labeling times than peroxidases − Assembly depends on the design of the expression system, including linker length and fragment orientation − Lower catalytic activity compared to full‐length AirID − Reduced activity below 37°C	Schaack et al. 2023
	Split‐APEX2	1 min	20 nm	25–37	− Higher temporal and spatial resolution compared to biotin‐ligases − Low background due to individual fragments being inactive − Does not require ATP	− Potentially toxic due to the use of H_2_O_2_ − Assembly depends on the design of the expression system, including linker length and fragment orientation − Lower catalytic activity compared to full‐length APEX2	Han et al. 2019; Lam et al. 2015
	Split‐HRP	1–5 min	20–50 nm	25–37	− Higher temporal and spatial resolution compared to biotin‐ligases − Low background due to individual fragments being inactive − Does not require ATP	− Potentially toxic due to the use of H_2_O_2_ − Assembly depends on the design of the expression system, including linker length and fragment orientation − Lower catalytic activity compared to full‐length HRP − Inactive in a reducing environment	Chen and Perrimon 2017; Martell et al. 2016
Laccase
LaccID	1–2 h	Undefined	37–58	− Uses a non‐toxic activating factor, oxygen (O_2_) − More compatible with live samples/sensitive experimental systems compared to peroxidases − Thermally stable − Plasma membrane‐specific activity	− Labeling radius is less well defined compared to HRP and APEX2 − Possible off‐target modifications due to the diffusion of reactive intermediates − New enzyme: few published studies for benchmarking − Limited applicability for intracellular PL	Lee et al. 2025
Ubiquitin‐like ligases	PUP‐IT	30 min–24 h	5 nm	37	− Uses a non‐toxic substrate − Low background and high specificity because PafA and Pup are absent in eukaryotic cells	− Reduced activity below 37°C − Large enzyme (PafA) − Strict requirement for direct physical enzyme‐substrate contact	Chen and Perrimon 2017
	PUP‐IT2	30 min	5 nm	37	− Uses a non‐toxic substrate − Reduced steric hindrance and non‐specific self‐labeling compared to PUP‐IT − Low background and high specificity because PafA and Pup are absent in eukaryotic cells	− Reduced activity below 37°C − Strict requirement for direct physical enzyme‐substrate contact	Yue et al. 2022
	NEDDylator	1 h	~5 nm	37	− Highly specific covalent tagging of proteins in direct physical contact with the enzyme − Low background compared to peroxidases or biotin‐ligases	− Multi‐step enzymatic cascade increases system complexity, potentially introducing variability in labeling efficiency − Strict requirement for direct physical enzyme‐substrate contact	Wang et al. 2023
Peptide‐based	Sortase A	10–30 min	1–2 nm	~37	− Highly specific labeling restricted to direct enzyme‐substrate interactions − Suitable for in vivo applications − Lower background compared to diffusion‐based systems	− Low efficiency: dependent on contact geometry, local protein density and interaction time − Requires extensive prior genetic engineering	Ge et al. 2019
Glycan‐based	FucoID	30 min	Few nm	~37	− Enables selective detection of cell–cell interactions − Higher spatial specificity than diffusion‐based approaches	− Efficiency depends on interaction geometry, local glycan density, and cell–cell interaction time, potentially missing transient or weak contacts − Requires careful enzyme/substrate controls to avoid non‐specific labeling or background	Qiu et al. 2022

*Note:* Key characteristics, advantages, and limitations are highlighted.

Abbreviations: APEX, ascorbate peroxidase; ATP, adenosine triphosphate; h, hours; H_2_O_2_, hydrogen peroxide; HRP, horseradish peroxidase; nm, nanometer.

TurboID and miniTurboID were developed to overcome the known limitations of BioID and BioID2 (Branon et al. 2018; Chen and Perrimon 2017). Derived from the same *E. coli* scaffold, these variants were extensively engineered to achieve substantially higher catalytic activity, while maintaining compatibility with live‐cell labeling. TurboID achieves robust biotinylation in 10 min, producing signal levels comparable to BioID. This makes TurboID one of the most widely used enzymes to study transient interactions and/or rapidly changing protein neighborhoods in cell‐based systems (Table 1), though its performance in vivo may be constrained by biotin availability and ATP‐dependent activity. It also displays strong activity across a broad temperature range and in diverse subcellular environments, from the cytosol to membrane‐bound organelles, enhancing its versatility for use across biological systems and model organisms. However, its high efficiency and elevated affinity for biotin can, in some circumstances, lead to non‐specific labeling or the depletion of cellular biotin (which can induce cell toxicity). MiniTurboID, a truncated variant lacking the N‐terminal domain of TurboID, is not only smaller (offering steric advantages) but also shows reduced background activity, albeit at the expense of lower overall labeling efficiency than full‐length TurboID (Branon et al. 2018). MicroID, microID2, and ultraID represent further refinements of this tool. MicroID is a truncated, less active version of TurboID, which shows reduced background biotinylation (Na et al. 2022). MicroID2 is an incremental improvement, which displays increased labeling efficiency while retaining low levels of background labeling, although it still does not reach the kinetics of full‐length TurboID (Johnson et al. 2022). In contrast, ultraID combines rapid kinetics with high specificity, enabling robust labeling within minutes, while minimizing non‐specific biotinylation. However, it is less widely adopted than TurboID, likely reflecting TurboID's earlier publication, the broader availability of validated constructs (e.g., from Addgene), and the practical advantage of cross‐study comparability, all factors which may be limiting its uptake in the field (Kubitz et al. 2022).

AirID and BASU expand the biotin‐ligase toolkit in new directions (Table 1). AirID was engineered to reduce cellular toxicity and off‐target labeling compared to TurboID, making it especially suitable for long‐term studies in sensitive cell types, but it still displays slower labeling kinetics than TurboID (Kido et al. 2020; Schaack et al. 2023). In contrast, BASU was developed primarily to probe RNA‐protein proximity, thereby extending PL applications beyond proteome mapping. Hence, its design makes it less suited for general protein–protein labeling. Moreover, in protein‐dense cellular environments such as fine glial processes, the lack of optimization for protein–protein interactions may increase background labeling, limiting its utility outside RNA‐centred applications (Ramanathan et al. 2018; Villaseñor et al. 2020).

The major advantage of biotin‐ligases is their compatibility with live‐cell labeling under physiological conditions, although the strict dependence on ATP may limit the range of applications the enzymes can be used for (e.g., intracellular versus extracellular labeling) (Wu et al. 2025). When applied to glial cell studies, biotin‐ligase‐based PL has proved a powerful tool to map local proteomes in fine cell processes and subcellular domains. Importantly, unlike peroxidase‐based systems, biotin‐ligases do not require hydrogen peroxide (H_2_O_2_), thereby avoiding the risk of oxidative stress and reactivity that could adversely impact glial physiology. This makes them particularly attractive for probing glial networks in their native, non‐reactive states. Nevertheless, careful optimization is essential to minimize background labeling and biotin depletion, ensuring reliable insights into glia‐specific proteomes.

### 2.2 Peroxidases

Peroxidase‐based PL relies on two distinct peroxidases, namely horseradish peroxidase (HRP) and ascorbate peroxidase (APEX) (Becker et al. 2023; Chen and Perrimon 2017; Lam et al. 2015; Martell et al. 2012). These enzymes use H_2_O_2_ to oxidize phenol‐based compounds, such as alkyne‐phenol and biotin‐phenol, generating highly reactive phenoxyl radicals, which react primarily with electron‐rich amino acid residues, such as tyrosines (Chen and Perrimon 2017). As these radicals generally have a very short half‐life it means they react only with proteins in extremely close proximity. In practical terms, this means these systems label nearby proteins with high precision (within ~20–50 nm) and in very short times (~1–10 min) (Table 1). In comparison to biotin‐ligase‐based systems (labeling radius ~10 nm; labeling kinetics of hours), the main advantage of peroxidase‐based systems clearly lies in their superior temporal resolution rather than spatial specificity (Chen and Perrimon 2017) (Figure 2B).

To streamline downstream processing, a number of biotin‐based derivatives have also been introduced, each with specific characteristics which collectively enhance the versatility and efficiency of peroxidase‐based labeling. For example, BxxP incorporates a longer linker connecting the biotin moiety to the phenolic group, which reduces steric hindrance during streptavidin binding and improves peptide recovery, compared to other biotin‐phenol probes with shorter linkers. Furthermore, the longer polyamide linker makes it membrane‐impermeant, so it is particularly suitable for selective labeling of extracellular proteins (Loh et al. 2016). Desthiobiotin‐phenol represents a further optimization of biotin‐based probes, in which the biotin tag is replaced by a desthiobiotin group, which has a lower affinity for streptavidin than standard biotin, allowing milder and reversible streptavidin‐based purification, facilitating protein elution and reducing background (Lee et al. 2017).

HRP, an enzyme traditionally used in immunohistochemistry (Ryan et al. 2006), has been adapted for PL and used in particular to study the cell surface proteome and/or extracellular matrix. Labeling occurs within minutes (~5–10 min) and does not require ATP (which is generally low in the extracellular space), enabling capture of transient protein networks in accessible compartments, where proteins can be rapidly tagged. However, the labeling procedure is generally much more involved than with TurboID, as HRP requires activation with H_2_O_2_, and so care must be taken to avoid introducing artifacts as a result of oxidative stress (Hollensworth et al. 2000). This can be done, for example, through the addition of quenchers (such as sodium azide) to rapidly decompose residual H_2_O_2_ and radical scavengers (such as sodium ascorbate) to neutralize reactive intermediates, both of which ensure complete termination of the labeling reaction. Finally, HRP has a limited range of applications, as its enzymatic activity depends on proper folding in a non‐reducing environment and on correct glycosylation (Škulj et al. 2022).

In contrast, APEX is an engineered ascorbate peroxidase that was originally developed for intracellular environments (Chen and Perrimon 2017; Martell et al. 2012). The improved version, APEX2, exhibits improved catalytic activity and has become a valuable tool for probing subcellular compartments, while minimizing oxidative stress. Recently, APEX3, an optimized version of APEX2, was developed to address limitations arising from biased subcellular localization and activity (Table 1) (Becker et al. 2023). Whereas APEX2 tends to accumulate in the cytoplasm and is sensitive to the nuclear export inhibitor leptomycin B (LMB), APEX3 incorporates a modified nuclear export signal (NES), achieving more balanced cytoplasmic and nuclear distribution. Moreover, APEX2 exhibits higher catalytic activity than HRP and a more restricted labeling radius (~20 nm compared with ~20–50 nm for HRP), which provides both improved temporal and spatial precision.

Taken together, therefore, these peroxidases are suited to distinct experimental contexts, with HRP preferentially used for labeling proteins at the cell surface and APEX2 preferred for subcellular labeling within the cytosol or organelles. However, while peroxidases offer unmatched temporal precision, their application in glial proteomics needs to be balanced with strategies that minimize oxidative perturbations.

### 2.3 Split Variants

**FIGURE 3:**
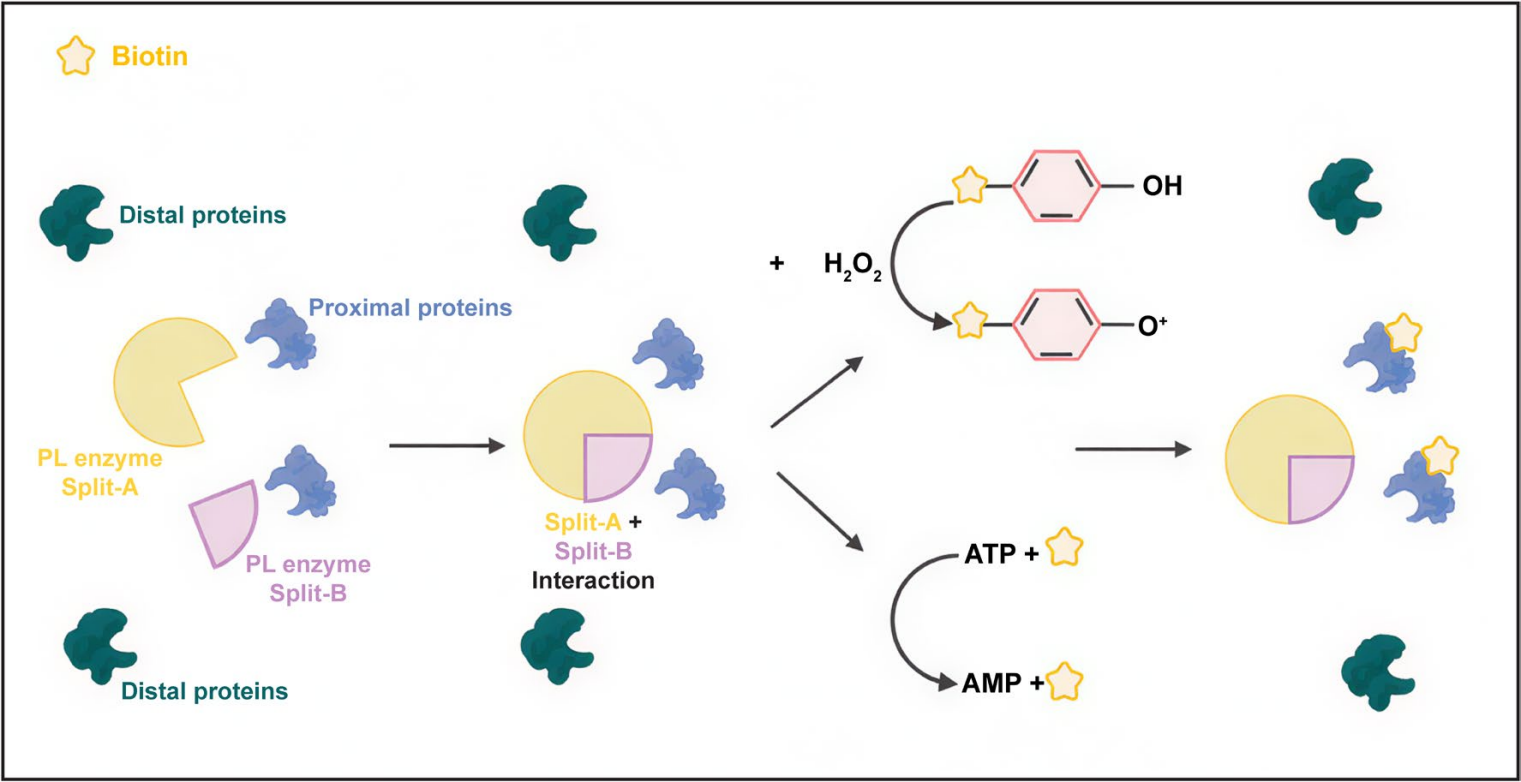
Schematic representation of proximity‐dependent protein labeling using a split enzyme system. The enzyme is divided into two inactive fragments that reassemble into an active form when brought into contact, enabling the covalent labeling of neighboring proteins, while distant ones remain unmodified. Created with BioRender.com.

Split‐enzyme PL represents a further refinement of the PL technique, introducing a greater degree of spatial control. In these systems, enzymes such as HRP (Martell et al. 2016), APEX2 (Han et al. 2019), BioID (De Munter et al. 2017; Schopp et al. 2017), TurboID (Cho et al. 2020), or AirID (Schaack et al. 2023) are divided into two catalytically inactive fragments that regain activity only when brought into close physical proximity (Figure 3)—for example when expressed on the surface of neurons and astrocytes at the tripartite synapse (Takano et al. 2020)—resulting in improving spatial selectivity and lower non‐specific labeling.

A key challenge of split‐enzyme systems is that their catalytic efficiency is generally reduced when compared to the full‐length enzymes. This may arise from multiple factors, including low affinity of the fragments for each other, entropic penalties associated with fragment reassociation, and structural perturbations that can compromise correct active‐site assembly. In addition, individual fragments may display reduced stability or solubility and may be partially misfolded in isolation, further limiting productive reconstitution under physiological conditions. Linker length and flexibility, as well as local molecular dynamics and alignment may also critically influence reassembly efficiency, which can vary substantially depending on the cellular context (Samavarchi‐Tehrani et al. 2020). To overcome perhaps the most common of these issues—low affinity interactions between the enzyme fragments—several strategies have been developed. These include chemically inducible dimerization systems, such as the rapamycin‐induced interaction of FK506 Binding Protein (FKBP) and FKBP–rapamycin binding (FRB) domains, which brings the fragments together in a controlled (and reversible) manner (Cho et al. 2020). Alternatively, enzyme fragments have been genetically fused to known pairs of cell adhesion proteins to promote enzyme assembly (Martell et al. 2016), as well as co‐targeting of fragments to specific membrane compartments using lipid anchors (Han et al. 2019). In addition, directing split fragments to defined subcellular locations or along specific trafficking pathways can further enhance their local density, favoring efficient enzymatic reconstitution and increasing labeling activity.

Overall, split‐enzyme PL approaches generally trade maximal labeling efficiency for enhanced specificity and contextual precision, making them valuable tools for probing protein interaction networks underlying spatially restricted signaling events in complex cellular systems.

### 2.4 Other Enzymes

Biotin‐ligase and peroxidase‐based approaches, particularly TurboID, HRP, and APEX2, remain the most widely adopted PL tools. Their continued predominance reflects a combination of favorable properties, including compact enzyme size, robust catalytic efficiency, well‐established and standardized protocols, and extensive benchmarking for mass spectrometry (MS)‐based data interpretation. Together, these features have enabled reproducible, scalable, and easily interpretable PL experiments, facilitating cross‐study comparisons and accelerating biological discovery.

However, beyond peroxidases and biotin‐ligases, several alternative enzymes have been engineered for PL. These tools include laccases, ubiquitin‐like ligases, peptide‐based and glycan‐based systems, each of which works through distinct mechanisms to covalently tag nearby proteins. Although less widely applied than the canonical enzymes, these systems expand the methodological repertoire, providing (complementary) advantages, such as stability, reduced background, and compatibility with live‐cell studies. Even though none of these approaches have been used to date to study glial cells, their unique features mean it cannot be excluded that they will find use in future applications, so they are briefly discussed below.

#### 2.4.1 Laccases

Conceptually, laccase‐based systems are related to biotin‐ligases and peroxidases in that they rely on the enzymatic generation of reactive intermediates that covalently tag proximal proteins. In laccase‐based approaches, the enzyme catalyzes the oxidation of phenolic substrates to produce reactive species capable of modifying nearby targets. As with other PL strategies, these intermediates must diffuse from the active site of the enzyme before reacting with surrounding proteins. However, differences in the half‐life and reactivity of the generated species influence spatial resolution, potentially increasing background labeling compared with systems in which reactive intermediates are more short‐lived or tightly confined (Table 1). This feature may, therefore, limit precision compared with PL strategies based on direct covalent transfer, such as ubiquitin‐like ligases, which are discussed below, or with tightly confined peroxidase‐mediated reactions.

LaccID, an optimized variant developed through directed evolution of a fungal multicopper oxidase, partially addresses these limitations. Unlike its predecessor, LaccID is selectively active at the plasma membrane of both living and fixed cells, a property attributed to its dependence on secretory‐pathway maturation (glycosylation, copper cofactor loading, and disulfide bond formation), which is unavailable in the cytosol, mitochondria, or ER lumen (Lee et al. 2025). This inherent surface restriction limits biotinylation to surface‐proximal proteins without requiring additional targeting strategies. Notably, it uses oxygen (O_2_) as the terminal electron acceptor rather than H_2_O_2_, reducing cytotoxicity while maintaining enzymatic activity. This feature makes laccase‐based PL more compatible with extended live‐cell experiments and sensitive cellular systems compared to peroxidase‐systems. Nevertheless, compared with canonical peroxidase‐based systems, such as HRP or APEX2, the effective labeling radius of LaccID may be less tightly constrained (at present it remains undefined), potentially resulting in increased background due to differences in intermediate stability and reactivity.

#### 2.4.2 Ubiquitin‐Like Ligases

Unlike traditional biotin‐ligase and peroxidase‐based systems, ubiquitin‐like ligases (including the so‐called NEDDylator system) are entirely genetically encoded, making them particularly well suited for in vivo applications, as they do not require the addition of exogenous substrates. These systems catalyse the covalent attachment of modifier proteins, such as ubiquitin or ubiquitin‐like proteins (UBLs), to lysine residues on substrate proteins. This reaction is a form of PTM in which the ligase is fused to a protein of interest, thereby exploiting this conjugation machinery to label proteins that directly engage with the “bait”. This is mechanistically distinct from TurboID or APEX2, which mark any protein within a defined spatial radius regardless of direct interaction (Wang et al. 2023). This requirement for direct enzyme‐substrate engagement, rather than simple spatial proximity, confers higher specificity and lower background compared with biotin‐ligase or peroxidase‐based systems, but typically results in reduced labeling coverage and efficiency. Furthermore, the reliance on engineered conjugation pathways and non‐standard capture tags/reagents has limited widespread adoption and standardization relative to TurboID‐ and APEX2‐based workflows (Wang et al. 2023) (Table 1).

In contrast to ubiquitin‐like ligase systems, PUP‐IT is a genetically encoded system that employs the prokaryotic ligase PafA fused to a protein of interest to covalently attach small prokaryotic ubiquitin‐like protein (Pup(E)) tags to lysines on nearby proteins. Because PafA and Pup(E) are absent in eukaryotic cells, this system operates orthogonally to endogenous ubiquitination pathways, resulting in low background and high specificity. These tagged proteins can then be captured via affinity purification, typically using antibodies or engineered binding domains that recognize Pup proteins. Unlike other PL systems, which depend on the diffusion of activated substrates, PUP‐IT keeps the activated Pup(E) bound to the enzyme. This restricts the labeling radius and promotes tagging only upon direct enzyme‐substrate engagement, improving the specificity to proteins that come into close physical proximity with the “bait” protein (Liu et al. 2018). Nevertheless, in comparison with biotin‐ligase and peroxidase systems, labeling efficiency is inherently lower, given its dependence on productive enzyme‐substrate engagement. Furthermore, the requirement for co‐expression and sufficient local concentrations of both components adds experimental complexity and may constrain temporal resolution compared with single‐enzyme systems, such as TurboID or APEX2 (Yue et al. 2022) (Table 1). Finally, the relatively large size of PafA (55 kDa) can interfere with “bait” protein function, alter subcellular localization, or promote self‐labeling artifacts, complicating data interpretation (Chen and Perrimon 2017). PUP‐IT2 was designed to address these specific limitations by fusing only the small Pup(E) (7 kDa) to the “bait” protein, while PafA is supplied separately. This configuration reduces steric hindrance and non‐specific self‐labeling, while maintaining proximity‐dependent tagging of interacting proteins (Yue et al. 2022).

#### 2.4.3 Peptide‐Based Enzymes

Sortase A‐based PL relies on a bacterial transpeptidation reaction in which sortase A recognizes an LPXTG‐containing peptide substrate (where X denotes any amino acid, most commonly glutamic acid, in the widely used LPETG motif), cleaving the peptide bond between the threonine and glycine residues to form an acyl‐enzyme intermediate. This intermediate is subsequently resolved by nucleophilic attack from the free N‐terminal amine of a glycine (or oligoglycine) residue on a nearby protein or peptide, leading to covalent ligation. Importantly, sortase A displays low catalytic activity in the absence of appropriately positioned substrates, such that random collisions rarely result in productive ligation. While this property confers exceptional specificity and minimal background compared with diffusion‐based PL systems, it also imposes important limitations. Labeling efficiency is inherently low and highly dependent on interaction geometry, local protein concentration, and residence time. Moreover, in many implementations, this approach requires prior genetic engineering of recognition motifs on proteins or suitable glycine acceptor sites and is, therefore, less scalable and less readily applicable to endogenous proteome‐wide analyses. As a result, many transient, weak, or spatially proximal (but non‐contacting) associations are likely to be missed (Ge et al. 2019).

#### 2.4.4 Glycan‐Based Enzymes

FucoID exploits fucosyltransferase (FT)‐mediated fucosyl‐biotinylation to label sites of cell–cell interaction. “Bait” cells displaying surface‐anchored FT transfer a biotin‐modified fucose substrate to physically contacting “prey” cells, thereby tagging them and allowing physical isolation. As the transfer only occurs across a very small radius, it requires direct cell–cell contact. This property confers FucoID with exceptional spatial selectivity compared with diffusion‐based PL approaches, such as TurboID or HRP/APEX, which label proteins within a defined radius regardless of physical engagement. However, this same requirement also imposes important constraints. By definition, labeling is limited to surface‐accessible glycoproteins at sites of sustained cell–cell contact, precluding detection of broader protein networks or intracellular components. In addition, efficient tagging depends on a sufficient duration of interaction to allow enzymatic transfer, meaning that very transient or low‐affinity contacts may be under‐represented. Consequently, FucoID provides a highly specialized readout of interface‐localized proximity, rather than a comprehensive map of interacting or neighboring proteins. Moreover, because labeling relies on engineered sugar donors and glycosylation pathways, careful substrate optimization is required to minimize off‐target modification and ensure specificity. Furthermore, downstream MS interpretation is less standardized than for biotin‐ligase or peroxidase‐based workflows.

In summary, these systems illustrate the expanding diversity of PL strategies beyond canonical biotin‐ligase and peroxidase‐based approaches. Although not yet applied in astrocytes, their complementary properties (particularly covalent stability, contact‐specificity and reduced background) highlight their potential as specialized tools for interrogating molecular interactions or structured microdomains. However, the fact that they (generally) require multiple components to be co‐expressed in cells, alongside their requirement for direct enzyme‐substrate contact and/or engineered recognition elements positions them as niche approaches that will likely complement, rather than replace, more generally accessible systems, such as TurboID, HRP and APEX2 (Qiu et al. 2022).

## 3 Applications of PL in Glial Cells

PL approaches have been applied across several glial cell types, but their use remains uneven across the field. This distribution likely reflects a combination of technical accessibility and the availability of cell‐type‐specific genetic tools, rather than biological relevance alone. Astrocytes, the most abundant glial cell in the mammalian brain, account for the majority of studies to date, as well as the most complex ones. Their extensive interactions with neurons, synapses and the vasculature, together with the need to resolve compartment‐specific proteomes, have made them particularly well suited to PL‐based strategies in vivo (Lee and Chung 2024).

By contrast, applications of PL in other glial populations, including RGCs, OPCs, oligodendrocytes, and microglia, remain comparatively limited. Studies in these cell types have largely relied on biotin‐ligase‐based systems, most commonly TurboID, and have been predominantly conducted in vitro, with no further validation. As a result, these applications currently serve as proof‐of‐principle demonstrations, rather than comprehensive maps of glial proteomes.

Across glial cell types, PL studies have majorly focused on intracellular proteomes, with only three studies to date applying PL extracellularly in astrocytes (Irala et al. 2024; Takano et al. 2020; Wu et al. 2025). This bias likely reflects both conceptual and technical considerations. Intracellular labeling is experimentally cleaner and less prone to “contamination” from neighboring cells: interpreting proximity signals at the cell surface is inherently complex, as physical adhesion and dense extracellular environments can confound the distinction between functional interactions and mere spatial apposition. Additionally, most of the published studies employed biotin‐ligases that require ATP for efficient labeling. In extracellular environments, ATP concentrations are typically below 1 μM (Dunwiddie et al. 1997), approximately 1000‐fold lower than those required for optimal TurboID activity (Shuster et al. 2022).

In the sections below, we review how PL has been implemented across distinct glial populations, highlighting both the insights gained and the cell‐type‐specific limitations that currently shape the field. The studies are presented with an increasing level of experimental complexity, ranging from descriptive proteomic analyses performed in vitro to in vivo approaches that incorporate targeted validation of individual protein candidates.

### 3.1 PL in OPCs

OPCs are widely distributed glial progenitors in the CNS. They receive direct synaptic inputs from neurons and respond to neuronal activity, which has been shown to regulate their proliferation and differentiation into myelinating oligodendrocytes. In addition, they also participate in neurovascular interactions and immune‐related processes, highlighting their functional heterogeneity within the CNS (Buchanan and Cheadle 2025; Gibson et al. 2014). While PL‐based studies in OPCs are still relatively rare, recent studies have now started to apply PL‐based strategies to characterize the molecular environment of these cells (Table 2).

**TABLE 2:** Overview of studies applying PL in glial cells.

Cell type	Paper	Enzyme	Cellular localization	Cell compartment	Brain region (or in vitro)	Study summary
OPCs	Hardt et al. 2023	BioID	Intracellular	Microtubules; cytosol; nucleus	In vitro	Various DCLK1‐BioID fusions were expressed in NIH/3T3 cells to identify putative interactors responsible for neural stem cell differentiation into OPCs. Data revealed phosphorylation‐dependent transitions between expression of short and long DCLK1 isoforms that shape the interactome and potentially shape OPC function.
Oligodendrocytes	Smirnova et al. 2023	TurboID	Intracellular	Plasma membrane; cytoplasm; nucleus	In vitro	A MBP‐TurboID fusion was expressed in HEK293T cells, identifying 34 potential MBP interactors involved in cell adhesion, vesicular trafficking and lipid metabolism/ferroptosis.
Microglia	Sunna et al. 2023	TurboID	Intracellular	Cytosol	In vitro	TurboID‐NES enabled robust, cell type–specific proteomic profiling of microglia, capturing core microglial pathways and dynamic inflammatory responses (LPS‐mediated).
	Bowen et al. 2024	TurboID		Plasma membrane; mitochondria; ER	In vitro	TurboID fused to the N‐terminus or C‐terminus of the potassium channel Kv1.3 and stably expressed in BV2 microglial cells allowed the identification of domain‐distinct interacting proteins, with the N‐terminus associated with protein processing and the C‐terminus involved in immune signaling. Microglia activation with LPS led to specific changes in the C‐terminal interactome. Functional assays confirmed a role for the C‐terminus of Kv1.3 in interferon‐mediated STAT1 activation.
RGCs	D'Arcy et al. 2023	BioID	Intracellular	Basal endfeet	Cortex	In vivo BioID was used to map the proteome of radial glia basal endfeet in embryonic mouse cortex, identifying MYH9 and MYH10 as key regulators of endfeet morphology and subsequent interneuron organization.
Astrocytes	Sun et al. 2022	TurboID	Intracellular	Cytosol	Cortex	Cytosolic TurboID was expressed in astrocytes (*Gfap* promoter) and neurons (*CaMKII* promoter) following AAV‐mediated delivery, revealing cell type‐specific proteomes, highlighting unique signaling pathways.
	Rayaprolu et al. 2022	TurboID		Cytosol	Cortex, hippocampus, striatum/thalamus, pons medulla, cerebellum	Used a Cre‐dependent cytosolic TurboID mouse line crossed to cell‐type‐specific, tamoxifen‐inducible Cre lines (*CaMKII*—neurons; *Aldh1l1*—astrocytes), enabling in vivo proteomics. Reveals cell‐type and region‐specific signaling pathways and possible vulnerabilities to neurological disease.
	Soto et al. 2023	BioID2		Plasma membrane; endfeet; fine processes	Striatum	Various BioID fusions were used to label astrocyte and neuron subcompartments (cytosol versus plasma membrane; astrocyte endfeet and perisynaptic processes), revealing subcompartmentalization of functions. High levels of SAPAP3 (an actin regulator) were found in both the astrocyte and neuronal plasma membrane compartments. SAPAP3 is associated with OCD, and astrocytic and neuronal expression of SAPAP3 was found to make distinct contributions to the generation of OCD‐like repetitive behaviors and anxiety.
	Hill et al. 2025	TurboID		Endfeet (cytosolic TurboID combined with vessel purification)	Cortex	Cytosolic TurboID was expressed in astrocytes using AAV‐based delivery (*Gfap* promoter) and combined with a novel blood vessel‐endfeet purification technique. Over 2000 proteins were identified as endfeet enriched, with putative roles in transport, metabolism, and neurovascular signaling.
	van Veen et al. 2026	TurboID		Endo−/lysosomal compartment	Cortex	LAMP1‐TurboID was expressed in astrocytes via AAV‐based delivery coupled to use of the *Gfap* promoter. Quantitative MS (compared to a cytosolic TurboID control) was used to confirm the in vivo endo−/lysosomal localization of ATP13A4, a P5B‐type ATPase enriched in astrocytes.
	Cooper et al. 2026	TurboID		Gap junctions	Whole brain	TurboID fused to the C‐terminus of CX43 and expressed in astrocytes under control of the *GfaABC1D* promoter was used to trace astrocyte gap junction networks in vivo. Biotinylated proteins transit through gap junctions into coupled astrocytes, enabling whole brain mapping of astrocyte network topology. Combined with tissue clearing and light‐sheet microscopy, this approach revealed multiple distinct astrocyte networks with selective regional connectivity, varying from local to long‐range bilateral networks.
	Irala et al. 2024	TurboID	Extracellular	ECM (PNNs and brain parenchyma)	Cortex; anterior cingulate cortex	TurboID fused to astrocyte‐secreted neurocan (via its C‐terminal ELS domain) identified synaptic interactors, including those with putative roles in SST^+^ inhibitory synapse formation.
	Takano et al. 2020	Split‐TurboID		Perisynaptic astrocyte processes and neurons	Cortex	Split‐TurboID fragments expressed at the plasma membrane (using GPI anchors) in astrocytes (*Gfap* promoter) and neurons (*hSyn* promoter) following AAV‐mediated delivery. Split‐TurboID identified NrCAM as an astrocyte protein that interacts transcellularly with neuronal NrCAM (which itself is coupled to postsynaptic GEPH), promoting inhibitory synapse formation and function.
	Wu et al. 2025	HRP		Cell surface of astrocytes and neurons	Striatum	A HRP‐PDGFR fusion protein was expressed at the plasma membrane of either neurons or astrocytes following AAV‐mediated delivery (using *hSyn* and *Gfap* promoters, respectively). The intersection between datasets defines the astrocyte‐neuron interactome responsible for synaptic signaling. Synaptic interactions are disrupted in a mouse model of HD and can be rescued by genetic attenuation of mutant huntingtin expression in neurons.

*Note:* Summary of cell‐type and subcellular compartment targeted, the enzyme used, the brain region analyzed, and the major findings.

Abbreviations: AAV, adeno‐associated virus; *Aldh1l1*, aldehyde dehydrogenase 1 family member L1; *CaMKII*, calcium–calmodulin (CaM)‐dependent protein kinase II; CX43, connexin 43; DCLK1, doublecortin‐like kinase 1; ECM, extracellular matrix; ELS, epidermal growth‐factor like, lectin‐like, and sushi; ER, endoplasmic reticulum; GEPH, gephyrin; *Gfap*, glial fibrillary acidic protein; GPI, glycosylphosphatidylinositol; HD, Huntington's disease; HRP, horseradish peroxidase; LPS, lipopolysaccharide; MYH9/10, myosin 9/10; NES, nuclear export signal; NrCAM, neuronal cell adhesion molecule; OCD, obsessive‐compulsive disorder; OPCs, oligodendrocyte precursor cells; PDGFR, platelet‐derived growth factor receptor; PNNs, perineuronal nets; RGCs, radial glia cells; SAPAP3, SAP90/PSD‐95‐associated protein 3; SST, somatostatin; STAT1, signal transducer and activator of transcription 1; SYN, synapsin.

Hardt et al. (2023) were interested in the role of doublecortin‐like kinase 1 (DCLK1) in the transition of pluripotent neural stem cells (NSCs) into OPCs and ultimately into mature oligodendrocytes. DCLK1 plays a major role in this process and is tightly regulated during differentiation, undergoing a gradual shift in expression from the short to the long isoform. This isoform switching is controlled by phosphorylation of the SP‐rich region, which actually inhibits proteolytic cleavage of the long isoform into the short form. The authors chose to use BioID in NIH/3T3 cells for their experiments. Not only do NIH/3T3 cells endogenously express both DCLK1 isoforms, they are a genetically tractable model and support robust expression of recombinant constructs. Using isoform‐specific DCLK1‐BioID fusions allowed the authors to identify distinct interactomes: DCLK1‐short was associated with nuclear and proliferation‐related proteins, whereas DCLK1‐long predominantly interacted with microtubule and cytoskeletal components. Although these findings may not fully recapitulate the molecular and cellular biology intrinsic to OPCs, they provide first insights into DCLK1 biology suggesting that isoform switching represents a molecular mechanism linking phosphorylation, subcellular localization, and protein interaction networks to cell state transitions during oligodendrocyte differentiation. Together, this study exemplifies how PL can be used to interrogate intracellular interaction networks, although further validation in primary OPC cultures or in vivo models would undoubtedly strengthen the biological relevance of these findings.

### 3.2 PL in Oligodendrocytes

Oligodendrocytes are specialized glial cells of the CNS, responsible for producing myelin, the multi‐layered membrane that ensheathes axons to ensure rapid and efficient nerve impulse conduction. Beyond their classical role, they provide essential metabolic support to neurons, regulate ion and water homeostasis, and actively contribute to neural circuit modulation and plasticity (Stadelmann et al. 2019). Dysfunction or loss of oligodendrocytes is associated with a range of neurological conditions, including multiple sclerosis and neurodegenerative disorders, underscoring their importance for both neural integrity and cognitive function (Chen et al. 2022; Duncan et al. 2021). Given the complexity of oligodendrocyte interactions with neurons and other glial cells, there is a critical need for advanced molecular tools to dissect their specific roles within the CNS (Table 2).

To illustrate the potential of PL for studying oligodendrocyte biology, Smirnova et al. (2023) applied TurboID to identify potential interactors of myelin basic protein (MBP), a key component of the myelin sheath. Expressed in human HEK293T cells, MBP‐TurboID efficiently biotinylated neighboring proteins in the plasma membrane and cytoplasm, which were subsequently enriched and identified via MS. By comparing MBP to control proteins (deaminated MBP with reduced basic charge (Musse et al. 2006) and p21), the study identified specific MBP interactors from non‐specific background biotinylation. A total of 297 proteins were detected, of which 34 were unique to MBP, including adhesion molecules, vesicular trafficking proteins and factors involved in lipid metabolism and ferroptosis. This work highlights the utility of TurboID‐based PL for revealing both canonical and non‐canonical protein networks centred on myelin, while the use of MBPCit, an analogue of the C8 isoform enriched in aggressive forms of multiple sclerosis, provided unique insights into myelin biology during disease. At the same time, however, it does need to be acknowledged that the use of HEK293T cells in this study represents an important limitation; not only do they lack the specialized membrane architecture and lipid‐enriched environment that shape MBP function in oligodendrocytes in vivo, but they potentially lack expression of key MBP interactors. As such, further in vivo studies will be required to fully capture MBP‐associated networks in their native membrane environment.

### 3.3 PL in Microglia

Microglia are the resident immune cells of the CNS, playing essential roles in brain development, homeostasis, and response to injury or disease. Beyond their classical immune functions, including surveillance, phagocytosis, and cytokine release, microglia also actively regulate synaptic plasticity, participating in synapse pruning and circuit refinement. Under homeostatic conditions, microglia exist in “surveillance” mode, continuously monitoring the brain parenchyma and supporting neuronal function. In response to pathological stimuli, however, they undergo state transitions ranging from pro‐inflammatory to neuroprotective phenotypes, and dysregulation of these transitions contributes to neurodegenerative and neuroinflammatory disorders (Colonna and Butovsky 2017; Wendimu and Hooks 2022). Understanding the molecular mechanisms that govern these state transitions and microglia interactions with neighboring cells, therefore, remains a central challenge in glial biology.

PL has emerged as a transformative approach to address this challenge. While early studies focused mainly on neurons and astrocytes, recent work has applied TurboID approaches to microglia (Table 2). However, progress in this cell type has been comparatively slow, largely due to technical challenges associated with efficient transfection and viral transduction (Ball et al. 2024). Consequently, studies to date have relied predominantly on in vitro systems, including microglial cell lines—which, while meaningful, do not fully capture the cellular complexity and environmental cues present in the brain. In this section, we discuss key studies that have used TurboID in these cells, illustrating how this approach is enhancing our understanding of their proteomic landscape in health and disease.

The first study was conducted by Sunna et al. (2023), who applied TurboID‐NES to murine BV2 microglial cells—with N2A neuroblastoma cells used for comparison. Stable expression of the cytosolic enzyme captured a substantial fraction of the microglial proteome under basal conditions, without overly altering cellular physiology, inflammatory responses, or mitochondrial respiration. In microglia, the labeled proteome was enriched for proteins associated with endo−/lysosomal pathways, vesicle trafficking, translation and signaling—reflecting key immune cell functions. Importantly, TurboID‐NES sensitively detected proteomic changes induced by inflammatory stimulation (lipopolysaccharide—LPS). These included upregulation of interferon‐response and antigen‐presentation pathways, oxidative stress and phagocytic processes, as well as canonical pro‐inflammatory markers, such as interleukin 1 alpha (IL1A) and immunoresponsive gene 1 (IRG1). Together these results demonstrate the ability of PL to capture dynamic microglial states in situ.

In a second study, Bowen et al. (2024) used TurboID fused to the potassium channel Kv1.3, a channel strongly upregulated in activated and disease‐associated microglia, to map its proximal interactome in BV2 microglia during the transition towards a disease‐associated state following Toll‐like receptor 4 (TLR4) activation by LPS. This TurboID‐based approach enabled domain‐resolved identification of Kv1.3‐associated proteins, revealing distinct interaction networks linked to channel trafficking and immune signaling. Notably, activation of inflammatory signaling selectively upregulated immune‐related interactors, and functional validation confirmed a role for Kv1.3 in interferon‐dependent STAT1 signaling. Alongside Sunna et al. (2023), this study reinforces the utility of TurboID‐based PL for dissecting dynamic, stimulus‐dependent protein interaction networks in microglia by uncovering a non‐canonical immune function for a specific ion channel.

### 3.4 PL in RGCs

RGCs are key neural stem and progenitor cells in the developing CNS, giving rise to neurons, astrocytes and OPCs (which subsequently differentiate into mature oligodendrocytes), while also guiding migrating neurons along their radial processes. In addition, they contribute to the regulation of neural progenitor maintenance and differentiation both through direct cell–cell interactions and the secretion of signaling molecules within the ventricular niche (Nelson et al. 2013; Yoon et al. 2008). Characterized by their elongated, bipolar morphology and specialized basal endfeet, they express markers of both neuroepithelial and astrocytic lineages, reflecting their versatile roles during neurogenesis and, in some contexts, in adult CNS repair (Arellano et al. 2021; D'Arcy and Silver 2020).

PL has emerged as a powerful approach to probe the molecular architecture of RGCs, particularly in subcellular compartments that are challenging to mechanically isolate (Table 2). To illustrate the potential of PL for studying RGC biology, D'Arcy et al. (2023) employed in vivo BioID‐based PL to characterize the proteome of radial glia basal endfeet in the embryonic mouse cortex. They were able to reveal a distinct proteome, prominently featuring the non‐muscle myosins MYH9 and MYH10. Functional analyses demonstrated that these myosins play non‐redundant roles in endfeet organization, with MYH9 regulating branching and structural complexity and MYH10 mediating adhesion to the basement membrane. Conditional deletion of these proteins led to non‐cell‐autonomous defects in interneuron number and positioning, linking the molecular composition of RGC endfeet to cortical circuit assembly. This study represents one of the most advanced applications of PL in glial cells to date and highlights the power of BioID‐based PL to resolve compartment‐specific molecular networks in glial cells.

### 3.5 PL in Astrocytes

Astrocytes are the most abundant glial cells in the brain, characterized by an irregular star‐shaped morphology and broad endfeet—features which enable extensive contacts with neurons, vasculature and other glial cells. They provide essential structural and metabolic support to neurons (Endo et al. 2022). Although their morphology and canonical functions have been well described (Oliveira et al. 2026), far less is known about the molecular mechanisms that govern their communication with surrounding cells.

Addressing this gap requires tools that can capture dynamic and spatially restricted protein networks with high precision. As such, PL has emerged as a powerful approach to meet this challenge, enabling the identification of compartment‐specific proteomes and molecular interactions in living cells. In the past 5 years, astrocytes have emerged as the main glial cell type investigated using PL‐based methods (Table 2). Notably, most studies have employed biotin‐ligases (BioID2 and TurboID), reflecting both the suitability of these enzymes for addressing intracellular and subcellular compartment‐specific questions and potential concerns about the sensitivity of astrocytes to the H_2_O_2_ required by peroxidase‐based systems for activation (Feeney et al. 2008). In the following sections, we first review intracellular PL approaches in astrocytes before discussing extracellular and synaptic interface‐focused strategies, which remain fewer in number and present additional conceptual and technical challenges. Each section highlights a different facet—from subcellular proteomes to intercellular pathways—demonstrating how PL is beginning to reshape our understanding of these versatile cells.

While PL was first applied in astrocytes to investigate astrocyte‐neuron interactions (Takano et al. 2020), most studies that followed were focused on intracellular labeling strategies to define global and compartment‐specific astrocytic proteomes. Initially, Sun et al. (2022) developed an adeno‐associated virus (AAV)‐based PL strategy to investigate cortical astrocyte proteomes in vivo. By driving TurboID expression under the *Gfap* promoter (Nolte et al. 2001), they achieved astrocyte specificity, while *CaMKII* was used as a neuronal promoter (Dittgen et al. 2004). This allowed the authors to obtain selective enrichment of proteins from the two cell types. This approach identified 672 astrocytic proteins, including canonical astrocytic markers such as vimentin (VIM), excitatory amino acid transporter 2 (EAAT2) and aquaporin 4 (AQP4), confirming the specificity of the method. Beyond validating known astrocytic proteins, their dataset provides a broad proteomic resource for uncovering signaling pathways and molecular interactions specific to astrocytes.

Building on this, Rayaprolu et al. (2022) adopted a complementary genetic approach by generating *Rosa26* ^*TurboID*^ mice. When crossed with cell‐type‐specific Cre driver lines, these mice enable TurboID expression in selected populations without relying on AAVs, which may exhibit leaky and variable levels of expression (Gleichman et al. 2023). The use of inducible CreER^T2^ lines helped mitigate potential off‐target labeling associated with constitutive Cre expression, including recombination in RGCs and their progeny (although this strategy does require tamoxifen administration, which limits temporal resolution and can complicate studies of early development, as tamoxifen exerts estrogen‐like effects that can interfere with developmental signaling pathways) (Ved et al. 2019). This approach identified 925 proteins enriched in astrocytes compared to neurons. While some of these proteins may represent core astrocytic markers expressed across multiple brain regions, others likely contribute to region‐specific proteomic signatures, highlighting molecular heterogeneity. Indeed, cortical and hippocampal astrocytes were enriched for proteins associated with synaptic signaling and plasticity, whereas cerebellar astrocytes displayed signatures linked to metabolic support.

Unfortunately, no comparison was made between the two studies, therefore the degree of overlap between the two complementary approaches remains unreported (Rayaprolu et al. 2022; Sun et al. 2022).

Together, however, these studies provide convergent evidence supporting the robustness of cytosolic TurboID for capturing global astrocytic proteomes in vivo. However, because both approaches rely on cytosolic enzyme expression, they are biased towards soluble intracellular proteins and likely under‐represent membrane‐associated or luminal proteins, motivating subsequent efforts to achieve subcellular resolution.

Building on this foundation, Soto et al. (2023) made a major advance in the field by achieving subcellular resolution with BioID2. By selectively targeting the plasma membrane, endfeet and fine processes of striatal astrocytes, they generated distinct proteomic maps for each compartment, providing insights into the molecular machinery responsible for astrocytic signaling and homeostasis. In this study, endfeet were operationally defined as distal astrocytic domains and were not specifically characterized based on vascular association. Notably, SAP90/PSD‐95‐associated protein 3 (SAPAP3), a protein genetically linked to obsessive‐compulsive disorder (OCD), was enriched in fine processes and endfeet. Functional experiments revealed that astrocytic SAPAP3 regulates actin cytoskeleton organization and is required for the stabilization of inhibitory synapses; its loss disrupts inhibitory synaptic architecture and elicits OCD‐like repetitive and anxiety‐related behaviors in mice. Moreover, neuronal SAPAP3 contributed through complementary but distinct mechanisms, underscoring the need for dual consideration of astrocytic and neuronal contributions to psychiatric phenotypes. Together, this study exemplifies how PL can move beyond descriptive proteomics to identify astrocyte‐specific molecular regulators, with direct consequences for synaptic organization and behavior.

In contrast to the molecular targeting previously used, in which BioID2 was fused to AQP4 (Soto et al. 2023), Hill et al. (2025) used TurboID expressed under the control of the astrocyte‐specific *GfaABC1D* promoter—a synthetic, truncated version of the *Gfap* promoter—and combined it with vascular isolation and liquid chromatography‐tandem MS (LC–MS/MS). This approach achieved highly specific recovery of endfeet proteins, while avoiding contamination from endothelial cells. The authors identified over 2000 proteins, including SAPAP3 (Soto et al. 2023) and a core set enriched in membrane‐anchoring complexes, transporters, ion channels and receptors. Comparative analyses revealed significant overlap with previous datasets, including the ones from Soto et al. (2023), alongside the identification of more than 800 additional proteins, including all 10 of the most well‐known endfeet markers (AQP4, DAG1, DMD, DTNA, SNTA1, GPR37I, HEPACAM, KCNJ10, MLC1, and SLC4A4), which had not been comprehensively captured in previous studies (Alonso‐Gardón et al. 2021; Kameyama et al. 2023; Soto et al. 2023; Stokum et al. 2021), illustrating the power of PL combined with subcellular fractionation methodologies.

In a recently accepted manuscript, van Veen et al. (2026) used TurboID fused to lysosomal‐associated membrane protein 1 (LAMP1) (a type I transmembrane protein present in lysosomal membranes) to confirm the endo‐/lysosomal localization of ATP13A4. AAVs carrying LAMP1‐TurboID (or a control cytosolic TurboID) under the control of the *GfaABC1D* promoter were injected into postnatal day 1 (P1) mice, enabling selective biotinylation of proteins within the endo−/lysosomal compartments of astrocytes. MS confirmed the enrichment of ATP13A4 in LAMP1‐positive compartments, validating its subcellular localization in an in vivo context. Beyond the PL experiments, the authors further demonstrated that loss of ATP13A4 leads to reduced astrocyte morphology, enhances astrocyte‐mediated excitatory synapse formation, and leads to delayed early postnatal neurodevelopment followed by mild, female‐biased behavioral alterations in adulthood. Together, this study illustrates how compartment‐targeted PL can serve not only as a discovery tool, but also as a strategy for resolving the subcellular localization of endogenous proteins in specific glial cell types in vivo.

In contrast to intracellular and compartment‐specific approaches, extracellular PL strategies aim to capture secreted proteins and cell‐surface interactions that mediate communication between astrocytes and other cells. Even though these approaches can be more laborious, due to the complexity of the extracellular environment and likely risk of artifacts, recent studies have begun to adapt PL tools to probe astrocyte‐derived extracellular cues and astrocyte‐neuron interaction pathways in vivo.

Irala et al. (2024) first identified NCAN as an astrocyte‐secreted regulator of inhibitory synapse formation through analysis of transcriptome data, followed by validation in an in vitro synapse assembly assay. Functional experiments demonstrated that the C‐terminal Epidermal growth‐factor like, Lectin‐like, and Sushi (ELS) domain is both necessary and sufficient to drive inhibitory synaptogenesis. Strikingly, this effect was highly selective, since the ELS fragment localized preferentially to somatostatin‐positive (SST^+^) synapses, and its deletion caused a marked reduction in SST^+^ contacts, while parvalbumin‐positive (PV^+^) synapses remained unaffected. To characterize the associated extracellular environment, a secreted form of TurboID was fused to the NCAN C‐terminus enabling the identification of 46 proximal proteins. However, it should be kept in mind that any newly synthesized TurboID fusion protein that is destined for secretion must first transit through the secretory pathway and, as a result, some level of intracellular biotinylation could occur, introducing the possibility of artefactual labeling and necessitating the incorporation of strict validation steps for PL identified proteins. In this instance, therefore, rather than serving as a discovery tool, PL functioned as a contextualization strategy, defining the molecular niche in which a previously validated synaptogenic protein operates. These findings highlight a remarkable level of specificity in astrocyte‐secreted signals, showing that distinct interneuron subtypes can be differentially regulated by glial factors. By coupling PL with advanced imaging and genetic models, the study uncovered a mechanism through which astrocytes refine GABAergic circuitry, suggesting that extracellular cues, such as NCAN, may represent therapeutic targets in disorders characterized by excitation/inhibition imbalance.

To study extracellular astrocyte‐neuron interactions, Takano et al. (2020) used a split‐TurboID to probe the extracellular proteome of PAPs and astrocyte‐neuron junctions in vivo. In cortex, this approach identified 118 proteins enriched at so‐called “tripartite synapses”, unveiling a highly specialized molecular composition. Among these, NrCAM was found to be expressed in astrocytes, where it localized to perisynaptic contacts. Functionally, it was required to regulate the extent of astrocytic process infiltration into the neuropil and maintain perisynaptic contacts with neurons. Mechanistically, astrocytic NrCAM interacted homophilically with neuronal NrCAM; this neuronal protein is in turn linked to the postsynaptic scaffolding protein gephyrin (GEPH), thereby suggesting a mechanism by which astrocytes promote inhibitory synaptogenesis and stabilize GABAergic transmission. Although a key paper in the timeline of PL use in astrocytes, this approach is not without potential caveats. First, extracellular ATP concentrations in the healthy brain are typically below the levels required for optimal TurboID activity (Dunwiddie et al. 1997). Second, when using split PL systems, forced protein complementation, such as when using enzyme fragments fused to (overexpressed) cell adhesion molecules, may result in non‐physiological cell interactions being stabilized and artefactual protein networks detected.

Addressing these limitations, Wu et al. (2025) performed a systematic comparison of cell‐surface biotinylation using different enzymes and found that HRP‐based labeling exhibited higher activity for cell‐surface proteins than TurboID, consistent with the unique constraints imposed on enzymatic activity by the extracellular environment. They then went on and used HRP‐mediated PL to capture the cell‐surface proteomes of both striatal astrocytes and neurons. This identified a shared set of 561 proteins enriched for adhesion molecules, transporters, ion channels, and receptors, reflecting a structurally and functionally integrated surface network between the two cell types, in which astrocytes actively contribute to synaptic organization and circuit‐level signaling. The study also demonstrated that this shared proteome is disrupted in a mouse model of Huntington's disease (HD), with the majority of alterations at the level of the neuronal surface proteome, although astrocytic components were also affected. Neuron‐specific repression of mutant huntingtin (HTT) restored many of these alterations, underscoring the non‐cell autonomous nature of many neurodegenerative conditions and the therapeutic potential of targeting astrocyte‐neuron interactions.

Beyond proteome mapping, PL enzymes are starting to be repurposed and used to answer different questions. In fact, Cooper et al. (2026) exploited PL to study astrocyte intercellular connectivity. They developed an astrocyte network tracer by fusing TurboID to the C‐terminus of connexin 43 (CX43), the main gap junction protein in astrocytes. By expressing TurboID within the gap junction vestibule, only small molecules primed for gap junction flux are biotinylated as they pass into neighboring (uninfected) astrocytes, thereby revealing the extent of gap junction‐mediated coupling. By using whole‐brain tissue clearing and light‐sheet microscopy to facilitate detection of biotin within the intact tissue, this approach revealed that multiple astrocyte networks cross the mouse brain, each selectively connecting distinct brain regions. Networks varied in size and organization, ranging from local, region‐confined networks to long‐range bilateral networks, and their regional connectivity patterns were largely distinct from known neuronal networks. This study exemplifies how PL can be adapted beyond its conventional role in proteome discovery, serving instead as a tool to trace the topology and plasticity of glial communication networks in vivo.

Together, these studies illustrate the rapid methodological progression from whole‐cell to subcompartment‐specific proteomics, and from descriptive maps to functional and behavioral insights. Strikingly, the study by Cooper and colleagues demonstrates that PL tools do not need to be confined to proteome identification; they can be repurposed as tracers of intercellular connectivity, opening an entirely new dimension for investigating glial networks in vivo. Hence, these studies establish PL as a powerful approach for studying astrocyte biology in health and disease, and they will undoubtedly provide more information in future.

## 4 Challenges and Future Perspectives

PL approaches are beginning to reshape how protein organization and intercellular communication are investigated in glial cells within intact neural environments. At the same time, their application highlights several unresolved limitations that are particularly pronounced in glial biology.

A major set of challenges arises from the tools themselves. The biochemical requirements of PL enzymes greatly impact their applicability across the whole range of experimental settings. Peroxidase‐based systems, such as APEX and HRP, require exogenous H_2_O_2_ to initiate labeling, which can induce oxidative stress and limit their use in vivo, or in particularly sensitive cellular contexts. Accordingly, studies discussed in this review that employ these enzymes typically rely on acute brain slices to enable efficient labeling (Wu et al. 2025). Recently developed alternatives, including LaccID, bypass the need for H_2_O_2_, thereby reducing the potential for oxidative toxicity (Lee et al. 2025). By contrast, biotin‐ligase‐based approaches avoid oxidative stress, but generally require longer labeling windows (Table 1), which can reduce temporal specificity and increase background labeling.

A further conceptual limitation of PL using biotin‐ligase and HRP‐type systems is that it reports spatial proximity, rather than direct physical interaction(s). Overexpression of these enzymes can perturb endogenous protein networks, underscoring the importance of careful experimental design and independent validation (Bolognesi and Lehner 2018). Split‐enzyme systems can improve spatial specificity by restricting labeling to sites of enzyme complementation. Even though not all enzymes have split versions, these methods hold promise for uncovering (dynamic) molecular events underlying glial function. Alternatively, other strategies with higher specificity can be employed, such as ubiquitin‐like ligases fused to a “bait” protein, which require direct interactions for substrate modification. However, these require a much more involved experimental workflow and give generally less efficient labeling. In future, integrating affinity purification coupled with MS (AP‐MS) and proximity‐dependent biotinylation methods may well be the answer and provide more comprehensive interactome mapping than with either AP‐ or PL‐based methods alone (Liu et al. 2020). Nevertheless, additional orthogonal validation experiments will generally be required to conclusively establish direct physical interactions between proteins, such as isothermal calorimetry measurements on purified recombinant proteins (Baranauskiene et al. 2019).

Beyond these conceptual considerations, practical technical bottlenecks also merit attention. From a technical standpoint, the MS workflow for PL‐based experiments has become increasingly streamlined. Detection of biotinylated proteins is now routinely achieved through streptavidin‐based enrichment followed by LC–MS/MS. As such, the principal technical bottlenecks in PL experiments lie upstream of the MS step. These include all aspects of enzyme expression and localization to ensure efficient and comprehensive labeling, through to eventual sample preparation and recovery of proteins in sufficient amounts for MS analysis. Careful optimization of these parameters is, therefore, critical to ensure reproducibility and data quality. Downstream, the discrimination of genuine proximal interactors from background—including endogenously biotinylated proteins such as mitochondrial carboxylases—requires appropriate experimental controls, such as catalytically dead enzyme fusions or (untargeted) cytosolic controls (Casha et al. 2026). The absence of standardized bioinformatic pipelines across the field further complicates cross‐study comparisons, and the choice of statistical thresholds can substantially influence the resulting protein lists. Network‐level interpretation of PL datasets, using tools such as pathway enrichment analyses or protein interaction databases, adds valuable biological context but should always be complemented by experimental validation. In fact, a closely related challenge lies in moving beyond descriptive proteomic inventories towards mechanistic and functional insight. PL experiments typically yield large lists of candidate proximal proteins, but proximity alone does not establish direct physical interaction or functional relevance. The most informative studies discussed in this review are those that have coupled proteomic discovery in astrocytes with orthogonal functional validation. For example, SAPAP3 regulates the organization of the actin cytoskeleton, as well as inhibitory synapse stability, with direct behavioral consequences (Soto et al. 2023); NrCAM is functionally required for perisynaptic process infiltration and GABAergic transmission (Takano et al. 2020). Such studies illustrate a broader principle: the value of PL data is maximized when candidate proteins are subjected to loss‐ or gain‐of‐function experiments, imaging‐based localization studies, or biochemical validation. Looking to the future, integrating PL datasets with complementary approaches, including transcriptomics, spatial proteomics, and electrophysiology, will be essential to bridge the gap between molecular maps and our functional understanding of glial biology (Casha et al. 2026).

Temporal resolution represents an additional constraint. Many PL strategies rely on prolonged enzyme expression and extended labeling windows, averaging transient or sequential molecular events (Branon et al. 2018). While this limitation is less critical when studying relatively stable conditions, such as adult homeostatic states, it becomes particularly restrictive in the context of discrete developmental windows or acute glial responses (to injury), where precise temporal control is essential. Emerging light‐activated strategies, such as PhoTag and PhoxID (Oslund et al. 2022; Takato et al. 2025), as well as calcium‐dependent approaches including Cal‐ID and CaST (Kim et al. 2024; Zhang et al. 2024), are beginning to address these limitations. Although these methods have not yet been applied to glial cells, we believe they hold particular promise. Rather than employing constitutively active biotinylation, these approaches will allow interrogation of molecular events occurring within defined physiological timeframes.

A second set of challenges stems from the complexity of glial biology. A major issue lies in resolving molecular interactions between closely related cell populations that co‐exist within the same anatomical region, such as heterogeneous astrocyte populations in the cortex (Batiuk et al. 2020; Boisvert et al. 2018; Chai et al. 2017; Clarke et al. 2018; John Lin et al. 2017; Karpf et al. 2022; Morel et al. 2017; Zeisel et al. 2015). Identification of cell‐subtype‐specific promoters and/or the use of intersectional strategies will likely be needed to circumvent this issue (Holt 2023; Luo et al. 2008). However, as current PL strategies lack single‐cell specificity, any proteome obtained will represent the population average, and aspects of cell‐to‐cell variability (which are apparent at the transcriptome level) will not be detected. In parallel, when combined with the new generation of BBB‐penetrant viral vectors (Rincon et al. 2018), it should be possible to enable flexible and efficient targeting of various PL constructs across brain regions and cell types using a single systemic vector injection (although it should be remembered that at present viral systems tend to have low rates of microglia transduction). Additionally, glial cells, such as astrocytes and microglia, rapidly transition between functional states in response to neuronal activity, injury or disease, yet (as discussed previously) many PL strategies are limited by prolonged labeling windows, averaging transient molecular events together across time.

Despite these challenges, the rapid expansion of the PL toolbox suggests that many current limitations are technical rather than fundamental. The development of enzymes with improved temporal control, reduced toxicity and compatibility with split designs, together with approaches tailored to specific PTMs, such as recently developed tools like GlycoID (Nelson et al. 2024), is likely to broaden the range of glial questions that can be addressed. These advances are particularly important because they will allow the study of rapid or transient interactions, minimize perturbation of glial physiology, enhance spatial precision, and enable the detection of specific biochemical modifications, rather than only general protein proximity.

Beyond cell‐type‐specific proteome mapping, the recent repurposing of TurboID to trace gap junction‐coupled astrocyte networks across the brain by Cooper et al. (2026) illustrates that the conceptual scope of PL in glial biology extends well beyond its original design, and it is likely that future innovative applications of PL enzymes will shed further light on important aspects of in vivo glial biology, which have been impossible to approach until now.

Overall, therefore, while PL does not yet provide a comprehensive solution for dissecting the protein networks underlying glial signaling and interactions across all spatial and temporal scales, continued methodological innovation and integrative experimental strategies position it as a powerful and increasingly central approach for investigating glial cell biology in both health and disease.

## Author Contributions

M.G.H. conceived the manuscript. J.B. and R.A. wrote the original draft and created the figures using BioRender. J.B., R.A., and L.F. contributed to the conceptual development of the manuscript and the compilation of the tables. J.B., R.A., L.F., and M.G.H. reviewed and edited the manuscript.

## Funding

This work was supported by fellowship 2024.01587.BD to J.B. from the Fundação Para a Ciência e Tecnologia (FCT), Portugal. R.A. and M.G.H. are supported by the European Union's Horizon 2020 Research and Innovation Programme under grant agreement no. 951923 (NCBio ERA Chair). M.G.H. also receives support under FCT grant agreement 2023.17564.ICDT.

## Conflicts of Interest

The authors declare no conflicts of interest.

## Data Availability

Data sharing considerations are not applicable to this article, as no datasets were generated or analyzed during the current study.
